# Red Blood Cells Preconditioned with Hemin Are Less Permissive to *Plasmodium* Invasion *In Vivo* and *In Vitro*

**DOI:** 10.1371/journal.pone.0140805

**Published:** 2015-10-14

**Authors:** Véronique Gaudreault, Jakob Wirbel, Armando Jardim, Petra Rohrbach, Tatiana Scorza

**Affiliations:** 1 Département des Sciences Biologiques, Université du Québec à Montréal, Montréal, Québec, Canada; 2 Institute of parasitology, McGill University, Montréal, Québec, Canada; Bernhard Nocht Institute for Tropical Medicine, GERMANY

## Abstract

Malaria is a parasitic disease that causes severe hemolytic anemia in *Plasmodium*-infected hosts, which results in the release and accumulation of oxidized heme (hemin). Although hemin impairs the establishment of *Plasmodium* immunity *in vitro* and *in vivo*, mice preconditioned with hemin develop lower parasitemia when challenged with *Plasmodium chabaudi adami* blood stage parasites. In order to understand the mechanism accounting for this resistance as well as the impact of hemin on eryptosis and plasma levels of scavenging hemopexin, red blood cells were labeled with biotin prior to hemin treatment and *P*. *c*. *adami* infection. This strategy allowed discriminating hemin-treated from d*e novo* generated red blood cells and to follow the infection within these two populations of cells. Fluorescence microscopy analysis of biotinylated-red blood cells revealed increased *P*. *c*. *adami* red blood cells selectivity and a decreased permissibility of hemin-conditioned red blood cells for parasite invasion. These effects were also apparent in *in vitro P*. *falciparum* cultures using hemin-preconditioned human red blood cells. Interestingly, hemin did not alter the turnover of red blood cells nor their replenishment during *in vivo* infection. Our results assign a function for hemin as a protective agent against high parasitemia, and suggest that the hemolytic nature of blood stage human malaria may be beneficial for the infected host.

## Introduction

Malaria is a mosquito-borne infectious disease with a human etiology dating back to the divergence between great apes and humans, approximately 5 million years ago [1, 2]. This long period of interactions allowed both *Plasmodium* and humans to co-evolve in order to reach a trade-off state in which less than 1% of infected patients die [3]. However, considering the high number of afflicted individuals (>200 000 000 per year), malaria remains one of the most deadly infectious disease of our era.

*Plasmodium* requires the interaction with 2 cell types within the human body for completion of the complex life cycle, and the pathogenesis of malaria takes place during the asexual reproduction of the parasites in red blood cells (RBCs). The lasting coexistence of *Plasmodium* and humans has led to natural positive selection of various inherited blood disorders in malarial endemic areas that protect against malaria morbidity and mortality [4–8]. Among these are β-thalassemia, sickle cell disease and glucose-6-phosphate dehydrogenase deficiency, which are pathologies of distinct origins sharing several features that include weakened RBC membranes, anemia and hemolysis [6, 7, 9].

Hemolytic anemia is a hallmark of malaria, being the primary clinical manifestation of the infection. The pathophysiology of malarial anemia is multifactorial and involves the lysis of RBCs concurrent to *Plasmodium* invasion and growth, decreased production of RBCs by the bone marrow and, to a greater extend, destruction of uninfected RBCs [10]. The prevalence of the latter contributes to the complexity of malarial anemia by preventing a possible correlation between parasite burden and the severity of RBC loss [11]. Numerous mechanisms have been proposed to explain the exacerbated removal of RBCs from circulation during malaria infection, e.g. unspecific phagocytosis [11], accelerated RBCs senescence [12], opsonisation by auto-antibodies [13] and reduced membrane deformability [14]. Nonetheless, anemia, one of the most grievous manifestations of malaria, is still not fully defined and seems related to complex factors. In addition to the common symptoms of anemia (fatigue, dizziness, weakness), hemolysis also causes the release of hemoglobin (Hgb) into the circulation. In the presence of reactive oxygen species (ROS), Hgb gets rapidly oxidized into unstable methemoglobin that releases its heme groups, which, in turn, are oxidized to hemin (HE), a liposoluble, inflammatory and cytotoxic molecule [15, 16].

Previous studies have provided evidences that oxidative damage promotes HE-dependent eryptosis *in vitro* at very low HE concentrations (3–5 μM) in the absence of the HE scavenger protein hemopexin [17, 18]. Many studies have expressed concerns about the deleterious effects of HE in the pathogenesis of malaria [19, 20], especially since plasma hemopexin is known to be depleted in severe human malarial infection [10, 21]. We have demonstrated a strong immune modulatory property of HE, manifested as decreased secretion of interleukin-12, sustained production of interleukin-10 by murine macrophages *in vitro* and blunted interferon-γ production by spleen cells *in vivo* [22, 23]. These effects are known to be related to enhanced parasitemia and inhibition of the crucial Th2 helper cells/Th1 sequential polarization required to promote protective immunity against malaria [24]. However, although we and others have demonstrated how HE can possibly impair anti-malarial adaptive immunity, HE-preconditioned mice are protected against high parasitemia when infected with *P*. *c*. *adami* blood-stage parasites [23]. The beneficial effect of HE on blood stage malaria may be concurrent to enhanced destruction of infected-RBCs (iRBCs) or to a reduced capacity for parasite invasion or differentiation in HE-treated RBCs. Considering the possible relationship between HE and the unexplained severity of malarial hemolytic anemia, as an inducer and/or as a naturally selected favorable consequence, it was of great interest to understand how HE prevents high parasitemia in mice, and to assess the relevance of HE pre-treatment on *P*. *falciparum* development, the most virulent of the human malaria parasites.

## Material and Methods

### Treatment of mice

All animal protocols were approved by the UQÀM Institutional Animal Care Committee (protocols #0313-775-0314 and 0314-R1-775-0315). In certain experiments, female BALB/c mice 7- to 10-weeks old (Charles River Laboratories, Canada) were subjected to a reversed 12 h light/dark cycle in a temperature-controlled room, 2 weeks before use. One milligram of sulfo-NHS-LC-Biotin (Thermo Scientific, Canada) was solubilized in 100 μl of saline physiologic solution (0.9%) and was injected intravenously (iv) into each mouse. Mice were then injected by the intraperitoneal (ip) route with 10 mg/kg of freshly prepared HE (porcine; Sigma-Aldrich, Canada) or saline (control) for 3 consecutive days. For this, a 25 mg/mL (38.3 mM) stock HE solution was prepared in 0.1 M NaOH, and further dissolved in saline solution. Following treatment, control and HE-treated mice were infected with 5x10^5^ *P*. *c*. *adami* DK-parasitized RBCs from a syngeneic donor mouse, by the ip route. Parasitemia was estimated daily on 500 RBCs from tail tip blood smears fixed in 100% methanol and stained with 0.4% Giemsa (Sigma-Aldrich).

### Hematological analysis

Daily Hgb concentrations were determined by collecting 2 μL of tail tip blood in 498 μL of Drabkin’s solution (Sigma-Aldrich). Absorbance was measured in duplicate (540 nm) with a microplate reader (BioTek; Eon spectrophotometer), and a standard curve was performed with rat Hgb (Sigma-Aldrich) to convert the values into g/dL. To assess the percentage of reticulocytes and biotinylated RBCs, whole blood cells were stained with FITC-labeled rat anti-mouse CD71 monoclonal antibody (BioLegend, Canada, clone R17217) and APC-labeled streptavidin (strep) (BioLegend) in PBS (HyClone, Thermo Scientific; w/o Mg^2+^ and Ca^2+^), and incubated for 30 min in the dark at 4°C. Cells were washed once before fluorescence-activated cell sorting (FACS) analysis. RBCs turnover before infection was established as the percentage of biotinylated RBCs, which was multiplied by the number of RBCs in blood and presented as a concentration (10^9^ RBCs/mL) for post-infection turnover. Cell counting was assessed in 1 μL of blood diluted in 1 mL of PBS, and carried out with the flow cytometer previously calibrated with a reference count bead.

### Plasma hemopexin quantification

Hemopexin quantification was performed by ELISA commercial kit (Kamiya Biomedical, USA, cat no. KT-345) according to the manudacturer’s specifications (dilution 1/20 000).

### Oxidative status and RBCs ageing analysis

Altered membrane phospholipid asymmetry and RBC ageing was evaluated with Annexin-V (Life Technologies, Canada) and anti-CD47 (BioLegend, clone miap301) staining. Briefly, 1 μL of blood was collected in 1 mL of PBS, and centrifuged. Pellet was resuspended in Annexin binding buffer (Life Technologies), stained with FITC-labeled Annexin-V, PE-labeled rat anti-mouse CD47 monoclonal antibody and streptavidin-APC, and incubated at 20°C, in the dark for 15 min. Cells were washed and resuspended in PBS prior to FACS analysis of geometric mean of fluorescence intensity (gmeanFI) relative to Annexin-V and CD47 in streptavidin positive cells. Intra-erythrocytic ROS were measured using a modification of the Amer method [25]. Briefly, 2 x 10^6^ RBCs/mL were incubated at 37°C for 15 min in the dark with streptavidin-APC and 2’,7’-dichlorofluorescein diacetate (DCFDA; Sigma-Aldrich) dissolved in methanol. Cells were washed in PBS and analyzed for gmeanFI of 2’,7’-dichlorofluorescein (DCF) within streptavidin positive cells. Positive controls for Annexin-V and CD47 binding, and ROS measurement were achieved with a 15 min cells treatment with 50 μM hydrogen peroxide prior to washing and labeling.

### Flow cytometry analysis

Quantitative FACS analysis was performed on a BD Accuri™ C6 flow cytometer with the BD Accuri C6 software (BD Biosciences, Canada). Fluorescence intensities were expressed in logarithmic mode, and 40, 000 intact cells were acquired for each sample. Positive fluorescence was defined by a comparison with unstained control samples, except for streptavidin positive cells, which were compared to stained un-biotinylated cells.

### Evaluation of *Plasmodium chabaudi adami* blood stage with DAPI staining and DIC microscopy

To study late schizogony (merozoites formation), parasites invasion (extra-erythrocytic merozoites) and ring-stages during infection, blood smears were performed on glass coverslips #1.5 (Fisherbrand), at day 6 and 7 post-infection. Blood slides were fixed in 100% methanol and stained with 1 μg/mL 4’,6-diamidino-2-phenylindole (DAPI) dihydrochloride (Sigma-Aldrich) in the dark for 30 min. Coverslips were washed in PBS, allowed to dry and mounted on microscope slides with 30% glycerol in PBS. Cells were imaged by differential interference contrast (DIC) and fluorescence microscopy (Nikon A1 confocal, plan Apochromat VC 60x, numerical aperture 1.4, λs oil immersion) and analyzed with NIS-Elements Viewer 4.20 imaging software (Nikon).

### Assessment of parasites RBCs selectivity

DAPI stained smears of ring-stage *P*. *c*. *adami* infection and Giemsa stained smears of *P*. *falciparum* were analyzed for RBCs containing more that 1 ring-shape parasite in >150 iRBCs. The selectivity index (SI) was calculated as described by Simpson and others [26], and was defined as the ratio of the observed number of multiple invaded RBCs to that expected from an unrestricted random invasion process (Poisson distribution).

### Confocal microscopy quantification of infection

*P*. *c*. *adami* parasitemia was monitored by confocal microscopy to discriminate residual (strep ^+^) from newly differentiated (strep^-^) infected and multiple infected RBCs and reticulocytes (CD71^+^). At peak infection, 2 μL of blood were fixed overnight with 1% paraformaldehyde, washed with PBS and stained with 1 μg/mL DAPI, anti-mouse CD71-FITC and APC-conjugated streptavidin for 30 min in the dark. RBCs were washed and pellet dissolved in 100 μL of PBS. Drops of RBCs suspension were settled on glass coverslips #1.5. RBCs were imaged by DIC and fluorescence microscopy (Nikon A1 confocal, plan Apochromat VC 60x, numerical aperture 1.4, λs oil immersion) and analyzed with NIS-Elements Viewer 4.20 imaging software (Nikon).

### *Plasmodium falciparum* culture and treatment

*P*. *falciparum* strain Dd2 was cultured continuously using a protocol modified from Trager and Jensen [27]. In ~5% hematocrit, parasites were held in RPMI medium (Life Technologies, USA) supplemented with gentamicin (20μg/mL) (Life Technologies), hypoxanthine (0.1mM) (BioShop, Canada) and 0.5% AlbuMAX (Life Technologies), and maintained at 37°C, 5% CO_2_, 3% O_2_ and 92% N_2_. Erythrocytes (A^+^ blood type) were obtained from Interstate Blood Bank (USA). Parasites growth was monitored daily using Giemsa stained thin blood smears, and parasitemia was adjusted when required. Synchrony at ring stage was achieved by incubating parasites with 5% D-sorbitol (Bioshop) at 37°C for 10 min before resuspending parasites in culture medium. For HE pre-treatment experiments, uninfected RBCs (3.33% in culture medium) were incubated with 50 μM HE for 18 h at 37°C in a water bath. After removal of HE containing medium, parasites were cultured in triplicate with ~3.33% hematocrit using regular culturing conditions (37°C, 5% CO2, 3% O2, and 92% N2), with a starting parasitemia of 0.25%. Media was changed and thin blood smears for parasite counting were prepared daily. Parasitemia and blood stages were recorded based on blood smear quantification.

## Results

### HE administration does not promote premature ageing or removal of RBCS *in vivo*

**Fig 1:**
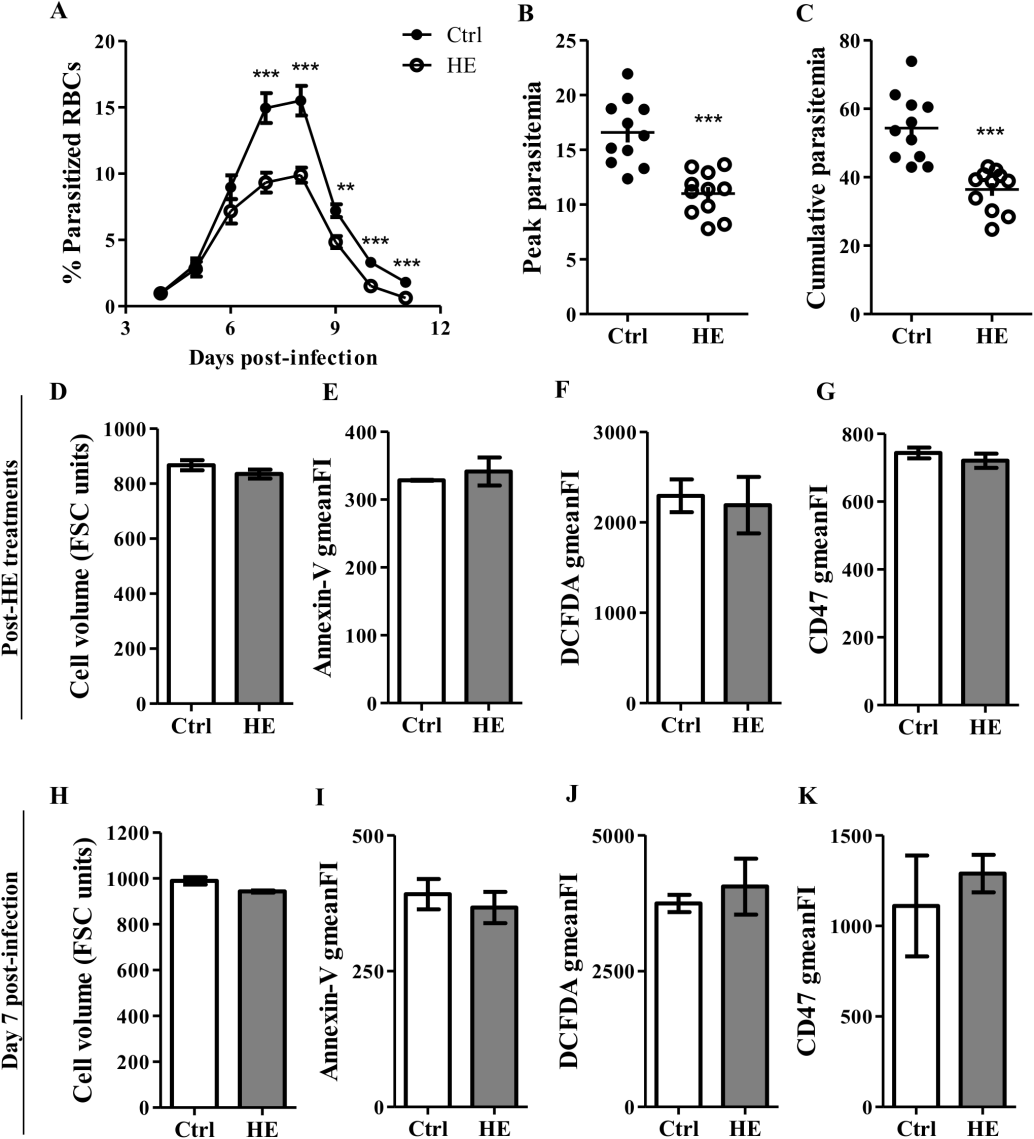
Markers of oxidative stress and ageing in RBCs from HE-preconditioned mice infected with *Plasmodium chabaudi adami*. BALB/c mice received intravenous injection of sulfo-NHS-LC-biotin (1 mg/100μl/mouse) to label RBCs in circulation, one day prior to a first HE treatment. Saline (control) or HE (10 mg/kg/day) were administered by the intraperitoneal (ip) route for 3 days and followed by ip injection of 5x10^5^ *P*. *c*. adami iRBCs, on the 4^th^ day. Tail-tip blood smears stained with Giemsa were performed daily to follow the parasitemia (A), determine the magnitude of peak parasitemia (B) and estimate cumulative parasitemia (calculated as the sum of daily parasitemia) (C). Three hours after the 3^rd^ and last saline/HE injection, the mean volume of RBCs was estimated from FSC values (D), and gmeanFI relative to Annexin-V binding (E), DCFDA labeling (F) and CD47 expression (G) were estimated by flow cytometry on 40 000 RBCs positive for streptavidin. These parameters were also analysed 7 days after *P*. *c*. *adami* infection (H-K). Data in A-C are the means ± SEM of three independent experiments (n = 11), and data in D-K are the means ± SEM of one experiment (Ctrl; n = 4, HE; n = 3). An unpaired Student *t* test was performed to compare the control group to the HE-treated one, *p<0.05, **p<0.01, *** p<0.001.

We previously reported decreased parasitemia in mice conditioned with HE prior to infection with *P*. *c*. *adami* DK parasites [23]. In an endeavor to study the effect of HE-preconditioning on RBCs subjected to *Plasmodium* infection, HE-treated RBCs were distinguished from RBCs differentiated post-treatment by sulfo-NHS-LC-biotin labelling *in vivo*, prior to HE injections, resulting in 98.1±0.9% labeled RBCs. Flow cytometry analysis included streptavidin staining to allow an exclusive comparison between cells in circulation during HE or saline treatment (strep^+^ cells), respectively in HE-conditioned and control mice. In the present investigation, the reported decreased peak and cumulative *P*. *c*. *adami* parasitemia in HE-preconditioned mice were reproduced, and an earlier recovery from the infection was also observed (Fig 1A–1C).

**Fig 2:**
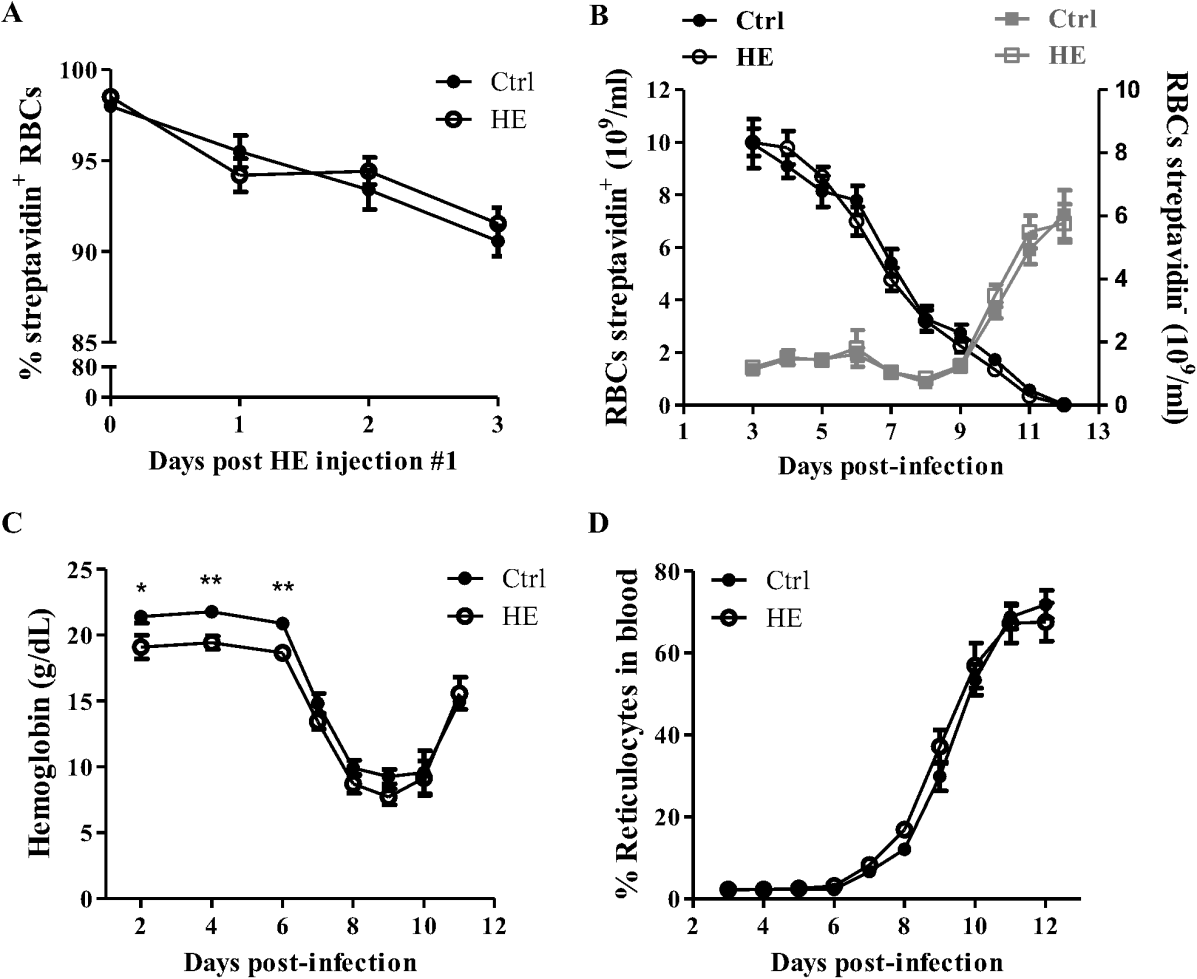
RBCs turnover in HE-treated infected mice. The percentage of sulfo-NHS-LC-biotinylated RBCs was followed by flow cytometry, starting 24 hours after the biotin injection, preceding HE injection (A). During the course of infection, RBCs turnover was estimated as the concentrations of biotinylated (strep^+^) and non-biotinylated (strep^-^) RBCs per mL of blood (B) by combining FACS streptavidin staining analysis with a flow cytometry blood cell count. Hemoglobin levels (C) and reticulocytes (positive gating of CD71^+^ cells with FACS) (D) were measured in whole blood. Results in A and D represent the means ± SEM of three independent experiments (A; n = 11, D; n = 7–11) and data in B and C are the means ± SEM of two experiment (n = 4–8). All data for saline and HE-conditioned mice were compared with an unpaired Student *t* test, *p<0.05, **p<0.01.

*Plasmodium* invasion and growth imposes stress on host RBCs [28, 29], and the possibility that HE-treated RBCs are removed from circulation prior to completion of parasite differentiation was addressed. To explore a plausible involvement of host RBC senescence and eryptosis in decreased parasitemia, the status of RBCs was evaluated 3 h after the last HE/saline injection, as well as 7 days post-infection. Hallmarks of eryptosis are cell shrinkage and membrane phospholipid scrambling, and enhanced intra-erytrocytic ROS activity and loss in expression of the CD47 membrane protein are features of oxidative stress and ageing in RBCs [17, 30, 31]. Cell volume was evaluated from forward scatter (FSC) FACS analysis and phosphatidylserine exposure at the surface of RBCs was estimated as the gmeanFI relative to Annexin-V binding. FACS analysis of control and HE-treated RBCs failed in disclosing any differences, suggesting that pre-treatment with HE does not decrease RBC volume or increase translocation of phosphatidylserine to the outer leaflet of RBC membranes (Fig 1D and 1E and 1H–1I). Similarly, assessment of intra-erythrocytic ROS activity and RBCs premature ageing, evaluated with the DCFDA and anti-CD47 antibody gmeanFI respectively, did not reveal major differences between control and HE-preconditioned RBCs, after HE treatment or during infection (Fig 1F, 1G, 1J and 1K). As oxidized or senescent RBCs are rapidly removed from circulation by phagocytes *in vivo*, RBC turnover was evaluated by combining the biotin labeling with flow cytometry blood cell counts. This strategy enabled in *vivo* estimation of the concentrations of new (strep^-^) and old (strep^+^) RBCs in the circulatory system. No significant differences were found in the concentrations of *de novo* generated and residual RBCs after HE treatment, or during the course of the infection (Fig 2A and 2B). However, as reported previously [23], lower concentrations of Hgb were measured in HE-preconditioned mice with respect to control mice prior to peak infection (Fig 2C). Concomitant with the faster parasitemia recovery in HE-preconditioned mice, the onset of reticulocytosis started earlier in these mice but, displayed an overall identical kinetic-curve as in control mice (Fig 2D). These results suggest that despite decreased parasitemia and lower Hgb levels in HE-preconditioned mice, HE does not result in an accelerated ageing and/or turnover of RBCs, prior to and post-infection.

### *Plasmodium chabaudi adami* schizogony is not altered, but merozoite invasion is delayed, in HE-treated RBCs

**Fig 3:**
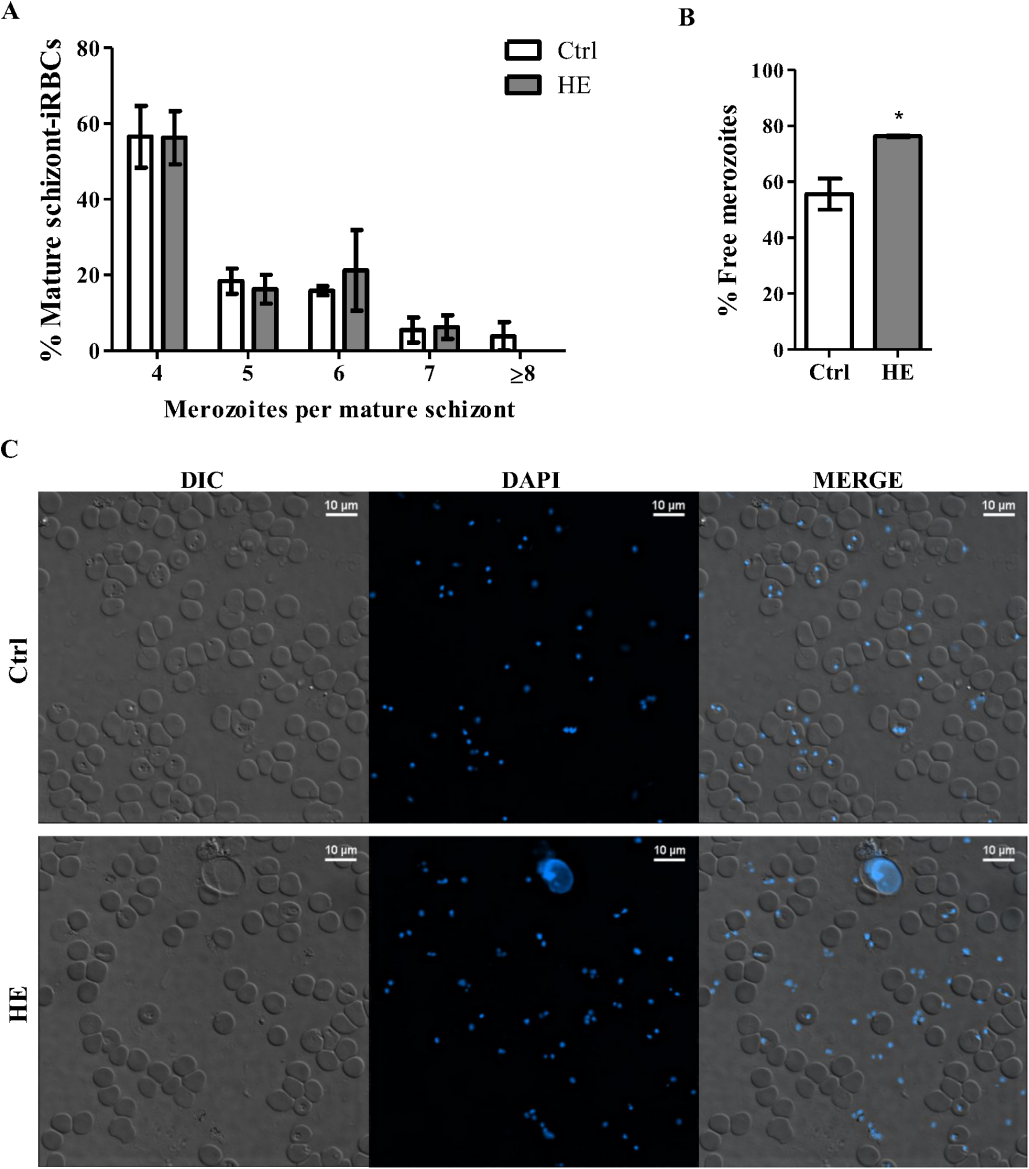
*Plasmodium chabaudi adami* schizogony and invasion stage. Six days post-infection, thin blood smears of iRBCs were prepared at the time of schizogony and stained with DAPI (blue). DIC and fluorescence images were captured with Nikon A1 confocal microscope (objective plan Apo VC 60x, NA 1.4, λs oil immersion), and analysed with NIS-Elements Viewer 4.20 imaging software. The proportion of merozoites (nuclei) per mature schizont (containing ≥4 merozoites) was determined in >150 iRBCs per mouse (A). DAPI-stained blood smears were also analysed at the time of merozoite invasion, and the proportion of extra-erythrocytic merozoites was measured in ≥600 merozoites (number of extra-erythrocytic merozoites/total number of merozoites) (B). DIC, DAPI fluorescence and merged images at the time of merozoite invasion are shown for a control and HE-treated mouse (C). The results represent the mean ± SEM of one experiment (Ctrl; n = 4, HE; n = 3) and were compared with an unpaired Student *t* test, *p<0.05.

Our data indicated reduced parasitemia in HE-conditioned mice without major changes in RBC turnover or premature ageing. Decreased parasitic replication concurrent with a reduced number of merozoites per schizogony cycle could account for the differences in parasitemia found in HE-conditioned mice. To evaluate a possible drop in merozoite numbers in HE-conditioned RBCs, a combination of fluorescence microscopy and differential interference contrast (Nomarski) imaging was adopted (S1 Fig). At the time of peak parasitemia, intra-erythrocytic merozoites were visualized within schizont-iRBCs as DAPI positive nuclei. The proportion of mature-schizont-iRBCs containing 4 or more merozoites did not differ between HE-treated and control mice (Fig 3A). However, DAPI-stained blood smears obtained from mice once the merozoite invasion cycle was accomplished (day 6 post-infection, 16 o’clock in a reverse light cycle) revealed higher numbers of extra-erythrocytic parasites in HE-preconditioned mice (Fig 3B and 3C). These data suggest that HE delays merozoite invasion of RBCs but does not affect schyzogony.

### The number of RBCs permissible to *Plasmodium chabaudi adami* invasion decreases in HE-conditioned mice

**Fig 4:**
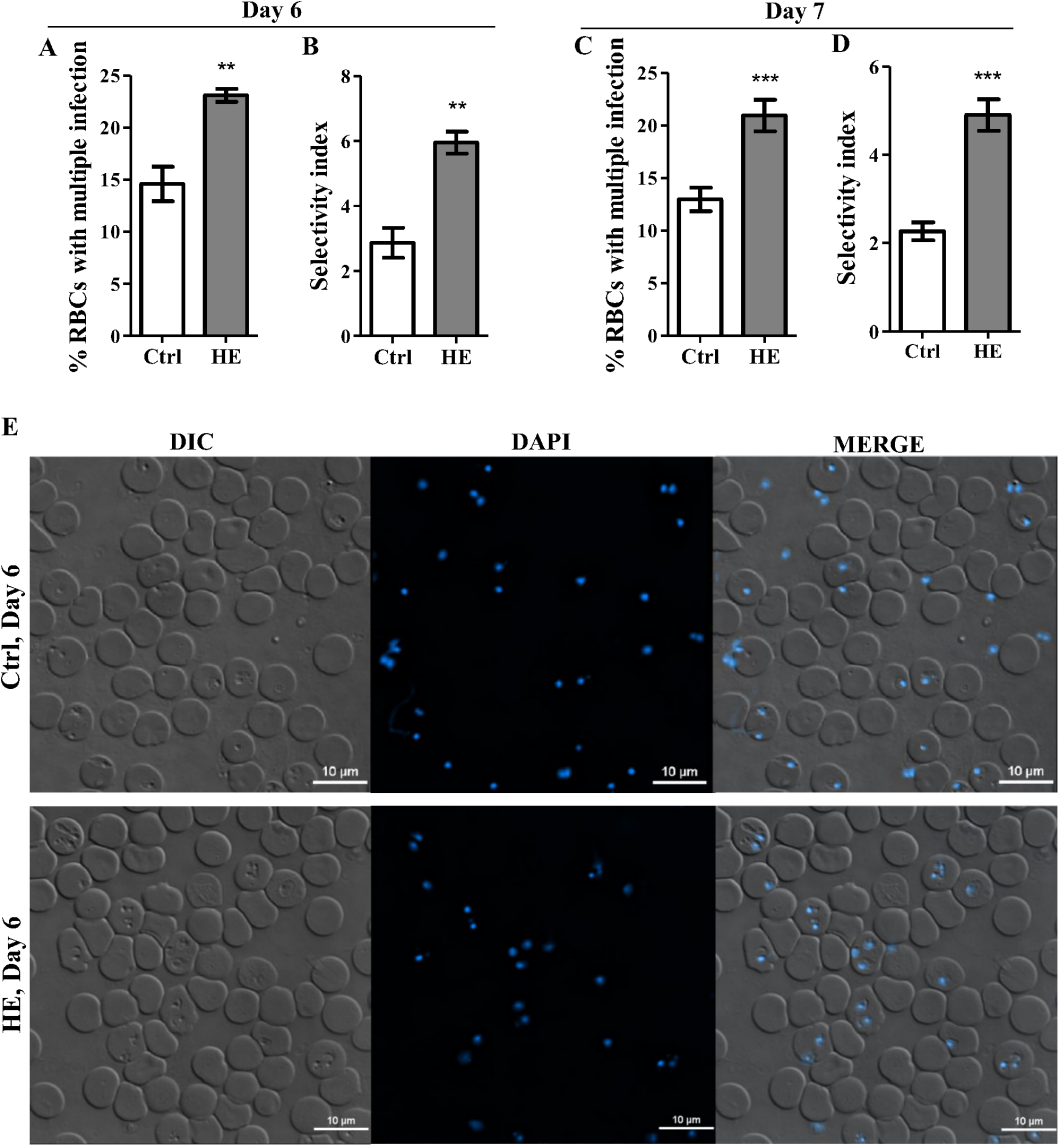
*Plasmodium chabaudi adami* selectivity for red blood cells. At day 6 and 7 post- *P*. *c*. *adami* infection, thin blood smears of parasite ring stages iRBCs were fixed and stained with DAPI (blue). DIC and fluorescence images were captured with Nikon A1 confocal microscope (plan Apo VC 60x, NA 1.4, λs oil immersion), and analysed with NIS-Elements Viewer 4.20 imaging software. Single and multiple-iRBCs were counted in >150 ring-iRBCs per mouse (A, C). Parasite SI was calculated for each day by dividing the observed value of multiple iRBCs by the value expected from a Poisson distribution (B, D). DIC, DAPI fluorescence, and merged channels are shown at day 6 post-infection during ring stage for a control and a HE-treated mouse (E). The results represent the means ± SEM of one experiment (Ctrl; n = 4, HE; n = 3) at day 6 post-infection and two experiments (Ctrl; n = 8, HE; n = 7) at day 7 post-infection. Data were compared with an unpaired Student *t* test, **p<0.01, ***p<0.001.

The severity of *Plasmodium* infections varies with the parasite strain and host RBC polymorphisms. Severe malaria and high parasitemia have been associated with unrestricted RBC invasion by the parasites [26, 32–34]. In contrast, uncomplicated malaria seems more related to selective RBC invasion e.g., *P*. *vivax* parasite’s preference for reticulocytes which results in lower (≤ 2%) parasite burden [26]. Our data revealed decreased parasitemia and limited invading capacity of merozoites in HE-preconditioned mice, suggesting that a more restricted RBC population is permissive to *P*. *c*. *adami* invasion and that merozoites may take more time to find suitable RBC to invade. From the analysis of DAPI-stained blood smears, the parasite selectivity index (SI) was estimated to establish a correlation between lower parasitemia and plausible more restricted RBC selection in HE-treated cells. This index was calculated by dividing the observed proportion of multiple-ring-iRBCs in control and HE-treated mice by the expected number of multiple-ring-iRBCs estimated from an unrestricted, or random, invasion process (Poisson distribution) [26]. The SI, at both 6 and 7 days post-infection, confirmed the data provided by fluorescence microscopy of DAPI-stained parasites rings-iRBCs, revealing a higher percentage of multiple-iRBCs in HE-preconditioned mice and more than 2 fold increase in *P*. *c*. *adami* RBCs selectivity (Fig 4).

### HE conditioned-RBCs impede *Plasmodium* invasion

**Fig 5:**
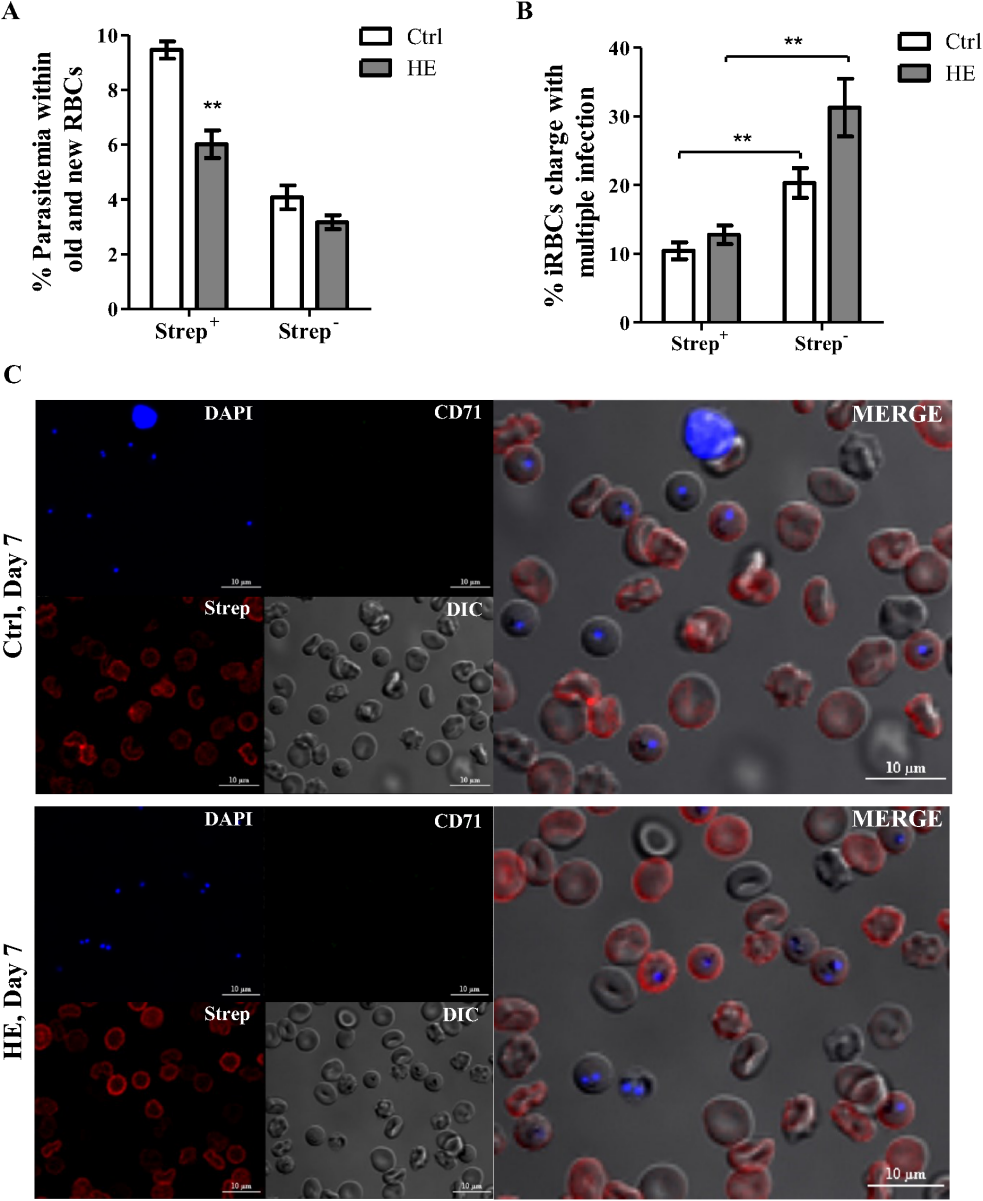
*Plasmodium chabaudi adami* parasites preferentially infect RBCs that have not been conditioned by HE. At peak parasitemia (day 7 post-infection), whole blood was fixed in 1% paraformaldehyde overnight, washed, stained and analyse with confocal microscopy. The parasites were labeled with DAPI (blue), and distinction between saline/HE-treated RBCs (strep^+^) and RBCs generated after treatments (strep^-^) was made by APC-conjugated streptavidin staining (red): reticulocytes were stained with FITC-labeled anti-CD71 antibody (green). DIC and fluorescence images were captured with Nikon A1 confocal microscope (plan Apo VC 60x, NA 1.4, λs oil immersion), and analysed with NIS-Elements Viewer 4.20 imaging software. Parasitemia within strep^+^ and strep^-^ RBCs (A; infected strep^+/-^ RBCs / total strep^+/-^ RBCs) and proportions of strep^+^ and strep^-^ RBCs charge with multiple ring (B; multiple infected strep^+/-^ RBCs / infected strep^+/-^ RBCs) were evaluated on >150 ring-iRBCs per mouse. DAPI, anti-CD71-FITC, streptavidin-APC fluorescence, DIC and merged channels are shown for a control and a HE-treated mouse (C). Data are the means ± SEM of one experiment (n = 4). All data from Ctrl and HE-treated mice were compared with an unpaired Student *t* test, **p<0.01, (Fig 5B; Ctrl strep^-^ vs HE strep^-^, p = 0.0593).

Considering the parasite SI estimated from HE-treated RBCs, the possibility that HE may directly influence the selectivity of RBCs by *Plasmodium* was evaluated. Using sulfo-NHS-LC-biotin labelling combined with streptavidin, DNA (DAPI) and reticulocytes (anti-CD71 antibody) staining, iRBCs conditioned by HE were distinguished from those generated thereafter. Confocal microscopy analysis revealed that the decreased parasitemia detected in HE-treated mice only occurred within the strep^+^ population when compared to control mice (Fig 5A). No major differences were found with respect to infection of new RBCs (strep^-^) between the two groups of mice. This observation suggests that the decreased parasitemia in HE-treated mice is concurrent with a direct effect of HE on RBCs that reduces their invasion by *Plasmodium*. It was also noticed that multiple infection preferentially occurred in strep^-^ for both groups of mice (Fig 5B). Considering that sulfo NHS-biotin labelling does not affect infection of RBC by *Plasmodium* [35], this observation rather suggests preference of *P*. *c*. *adami* for relatively younger but mature RBCs. In addition, the fact that hemopexin levels increased rapidly in both groups of *P*. *c*. *adami-*infected mice implies effective scavenging of free HE concurrent to hemolysis in this mouse infection model (S2 Fig).

### HE pre-treatment of RBCs reduces *Plasmodium falciparum* parasitemia and decreases the proportion of RBCs susceptible to parasite invasion

**Fig 6:**
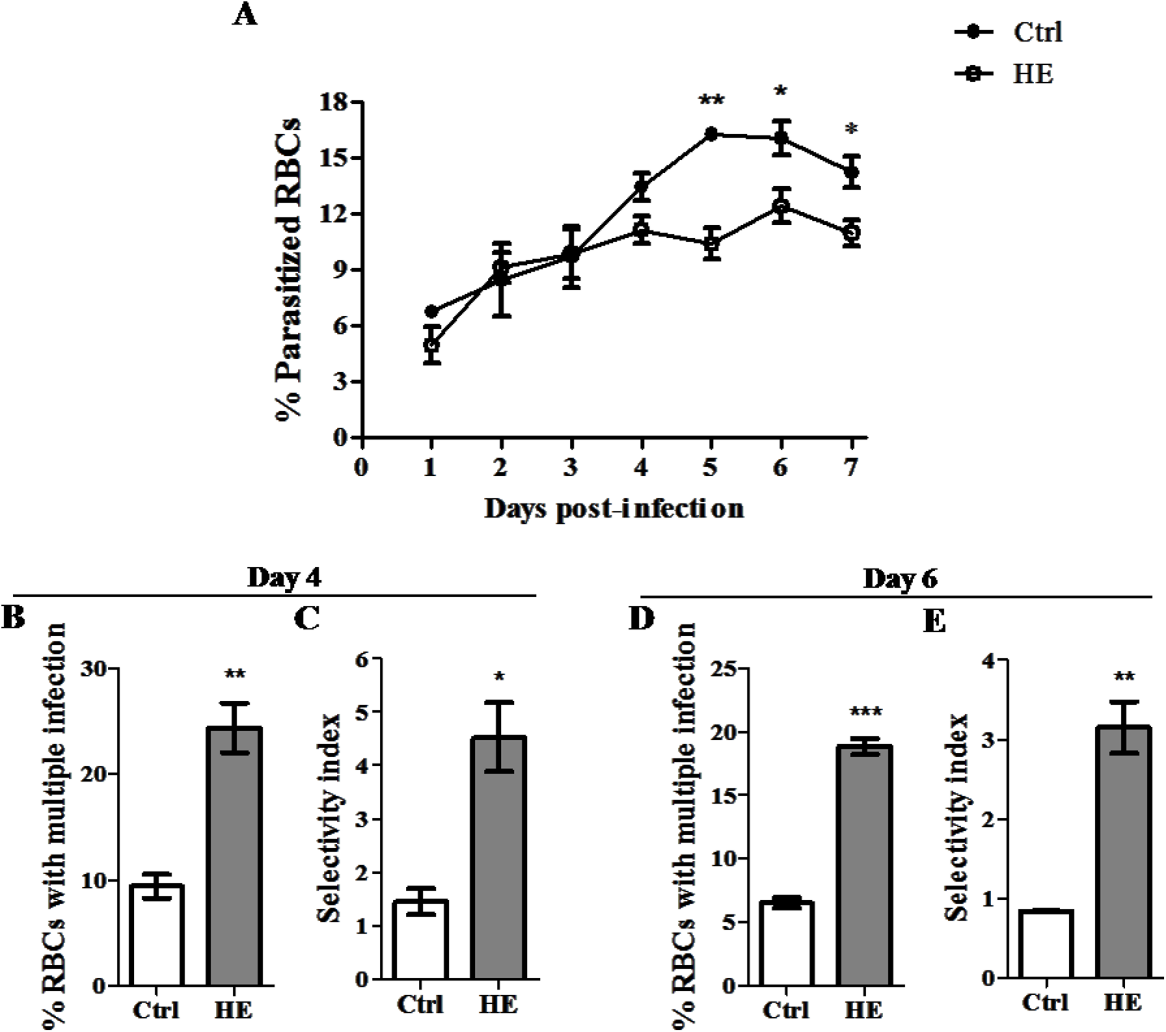
*Plasmodium falciparum* parasitemia and selectivity in HE-pretreated RBCs. Human RBCs were treated with saline/HE for 18 h and added to a culture of *P*. *falciparum* Dd2 parasites. Parasitemia was estimated from Giemsa-stained smears analysis (A), as was the percentage of multiple-iRBCs (B, D) and the SI (C, E), 4 and 6 days post-infection (during ring stage). The results are the means ± SEM of three independent experiments (n = 3), and data from Ctrl and HE-pretreated RBCs were compared with an unpaired Student *t* test, *p<0.05, **p<0.01, *** p<0.001.

To further establish a link between decreased *Plasmodium* parasitemia and HE, an assay was performed with *P*. *falciparum* parasites cultured in control or HE-pretreated RBCs. To avoid HE-induced eryptosis *in vitro*, RBCs were kept in medium containing albumin, a protein known to inhibit ~50% of HE-peroxidative activity [36]. Parasitemia was measured daily with Giemsa-stained smears and an SI was estimated at the time near to peak parasitemia (4 and 6 days post-*in vitro* infection). As with the *P*. *c*. *adami* infection in mice, *P*. *falciparum* parasitemia was significantly reduced from day 5 to 7 post-infection in HE-pretreated RBCs (Fig 6A). The reduced parasitemia was accompanied by significant increase in the percentages of iRBCs with multiple rings, and a 3 fold increase in *P*. *falciparum* RBCs selectivity index (Fig 6B and 6E). Thus, the observations with the mouse model were reproduced in the human infection model and suggest similar effects of HE on murine and human RBCs.

## Discussion

Severe hemolytic anemia in malaria is caused by the destruction of host RBCs following *Plasmodium* parasite growth and, to a greater extent, by the elimination of uninfected RBCs [10, 12, 37]. Free Hgb oxidises through ROS, resulting in methemoglobin formation and release of free unbound HE groups [16]. It has been reported that free HE exerts potent pro-oxidant, pro-apoptotic and pro-inflammatory properties, and that it controls a variety of biochemical processes involved in immune modulation [22, 23, 38–42]. Having previously reported that HE alters macrophages and T cells responses in a manner expected to be detrimental for the control of malaria [22, 23], it was surprising to find a decrease in *P*. *c*. *adami* parasitemia in mice preconditioned with HE. Our results suggest that the protection provided by HE against the development of high parasitemia in mice is not due to an increased elimination of iRBCs, nor to impaired merozoite differentiation. The higher prevalence of free merozoites as well as the increased selectivity imposed on the parasites, with reduced invasion capability of HE conditioned RBCs, suggests that HE renders certain RBCs less suitable to parasite invasion. Thus, the fact that a population of HE-preconditioned RBCs is still permissive to *Plasmodium* invasion indicates that not all RBCs are affected by HE in a comparable manner. Considering that RBCs progressively accumulate free HE in their membranes with age [43] and are attained by more oxidative insults [44–46], we speculate that older RBCs may be more sensitive to HE. This assumption may explain why *P*. *berghei* parasitemia is not affected in HE conditioned mice, as *P*. *berghei* parasites preferentially invade reticulocytes [47].

Rapid invasion of RBCs is fundamental for *Plasmodium* parasites, which are exposed to the host immune response during this transitory period, i.e., antibody opsonisation, cytotoxic, natural killer and γδ T cells killing, and macrophages and neutrophils phagocytosis [48, 49]. Time-lapse imaging analysis of invasion of RBC by *P*. *falciparum* and *P*. *yoelii in vitro* reveals an invasion capability in merozoites of only few seconds [50], which implies that not only the host immune response forces the merozoites to haste themselves into RBCs, but that other environmental or intrinsic factors (found *in vitro*) may exert pressure towards a quick invasion process. Accordingly, delayed invasion of HE-conditioned RBCs by *P*. *c*. *adami* merozoites, instigated by a reduced availability of permissive host cells, could be a possible cause for the decreased parasitemia found in HE-preconditioned mice.

The invasion process represents a rapid succession of steps involving the association and reorganization of several parasite/RBC proteins [50, 51], and any flaw of progression could result in reduced invasion by *Plasmodium*. Of the known impacts that HE may have on RBCs, the oxidative insults are potentially the most plausible causes for reduced infection [52]. The drop in parasitemia evidenced in *P*. *falciparum* cultures with HE-conditioned RBCs *in vitro* supports this idea. HE may cause peroxidation of membrane lipids and proteins [53],which are critical for merozoites invasion. Furthermore, HE induces conformational modifications of cytoskeletal proteins in RBCs [53, 54] that are essential for *Plasmodium* entrance [55, 56]. As the parasitophorous vacuole of *Plasmodium* is mostly composed of the host cell membrane lipids [57], it is reasonable to assume that peroxidation of these lipids may interfere with vacuole formation and parasite invasion.

The increased *P*. *c*. *adami* and *P*. *falciparum* SI found in HE conditioned mice and HE-treated RBCs cultures raises another plausible explanation for the decreased parasitemia following HE treatment. The SI predicts the specificity of *Plasmodium’s* merozoites for a particular population of RBCs. As the permissive population narrows, the chances for multiple merozoites invading a single RBC increase and a higher SI is expected. A SI of 2.5, as found for *P*. *c*. *adami*, indicates a nearly twofold increase in multiple iRBCs than predicted in a random distribution, and a mean SI of 1, as found in *in vitro P*. *falciparum* iRBCs, indicates a proportion of multiple iRBCs depictive of an unrestricted invasion process [26, 33, 34]. HE preconditioning increased these SI to 5.5 and 3.8, respectively for *P*. *c*. *adami* and *P*. *falciparum* infections, revealing additional two- and threefold higher chances that more than 1 merozoite infects a single RBC. The fate of such multiple iRBC is not known. It is acknowledge that the viability of the parasite may be hindered by nutritional and space limitations in face of multiple infections, and may represent a "dead end" for the parasite [58]. As mature schizonts enclosing higher numbers of merozoites were not evident in HE-pretreated infected mice, we may assume restricted individual development of the merozoites infecting a single RBC. Accordingly, the drop in parasitemia measured in HE-conditioned mice seems consequent to extra-erythrocytic neutralization of merozoites that fail in rapidly invading the permissive RBCs, and in limitation of *Plasmodium* development in multiple iRBCs.

Although studies have reported HE pro-eryptotic properties *in vitro* in absence of scavenger proteins [17, 18], our *in vivo* data suggest no impact of free HE on the viability of circulating RBCs and their turnover at the concentration tested, despite 60% drops in plasma hemopexin levels. Discrepancies between the *in vitro* and *in vivo* effects of HE may reflect the presence and recycling ability of hemopexin following its delivery of HE to hepatocytes, which supports the prevalence of unbound hemopexin and a persistent HE scavenging activity *in vivo* [59, 60]. The significant increase in plasma hemopexin found in *P*. *c*. *adami* infected mice suggests a difference in its induction pathway in mice and humans, since pronounced exhaustion of hemopexin occurs during human malaria [10]. In this respect, it is tempting to speculate that hemopexin depletion in the human host may further enhance the impact of HE on parasitemia, and thus may explain the disparity between severe malaria defined by parasitemias of ≈ 20% in mice versus ≥ 0.5% in man [61, 62].

One of the major outcomes in blood stage malaria is an exacerbated hemolytic anemia, which may be concurrent to various mechanisms [10, 12–14, 63, 64]. As a consequence, high plasma concentrations of free HE (≈ 20 μM) are reached in individuals with symptomatic malaria, and compromised hemopexin scavenging protection [21]. Similarly, many inherited blood disorders are characterized by chronic intravascular hemolysis of diverse aetiologies and heighten plasma concentrations of free HE. Interestingly, these disorders share a common protection against malaria morbidity and mortality [4–6]. According to the acknowledged Haldane’s malaria hypothesis [8], infectious diseases are a driving force in natural selection, and the long coexistence of humans and *Plasmodium* parasites has shaped the human genetic background in malaria endemic areas. In this respect, it is interesting to allege that severe malarial hemolytic anemia, as well as the protection provided by congenital hemolytic disorders, could in part arise from selective pressure tending towards the survival advantage of reduced life-threatening parasite densities concurrent to the release of HE.

## Supporting Information

S1 Fig *Plasmodium chabaudi adami* blood stage parasites imaging. Blood smears of iRBCs were performed during merozoite development through ring, trophozoite and late schizont. Smears were fixed with 100% methanol, stained with DAPI and imaged by fluorescence microscopy and differential interference contrast with a Nikon A1 confocal microscope (objective plan Apo VC 60x, NA 1.4, λs oil immersion), and analyzed with NIS-Elements Viewer 4.20 imaging software.
(TIF)

S2 Fig Hemopexin concentrations in the plasma of control and HE-conditioned mice during infection. Plasmatic hemopexin levels were quantified by ELISA, 24 hours after the third saline/HE treatment, as well as throughout the infection. The results are the means ± SEM of two independent experiments (n = 3–6), and were compared with an unpaired (Ctrl/HE) and paired (Ctrl day 0/day 7; HE day 0/day4) Student *t* test, ** p<0.01, *** p<0.001.
(TIF)
