# Supplementary figures and images for: Red Blood Cells Preconditioned with Hemin Are Less Permissive to Plasmodium Invasion In Vivo and In Vitro
Source: PLoS One. 2015 Oct 14;10(10):e0140805. doi: 10.1371/journal.pone.0140805 (PMC4605744; doi:10.1371/journal.pone.0140805)

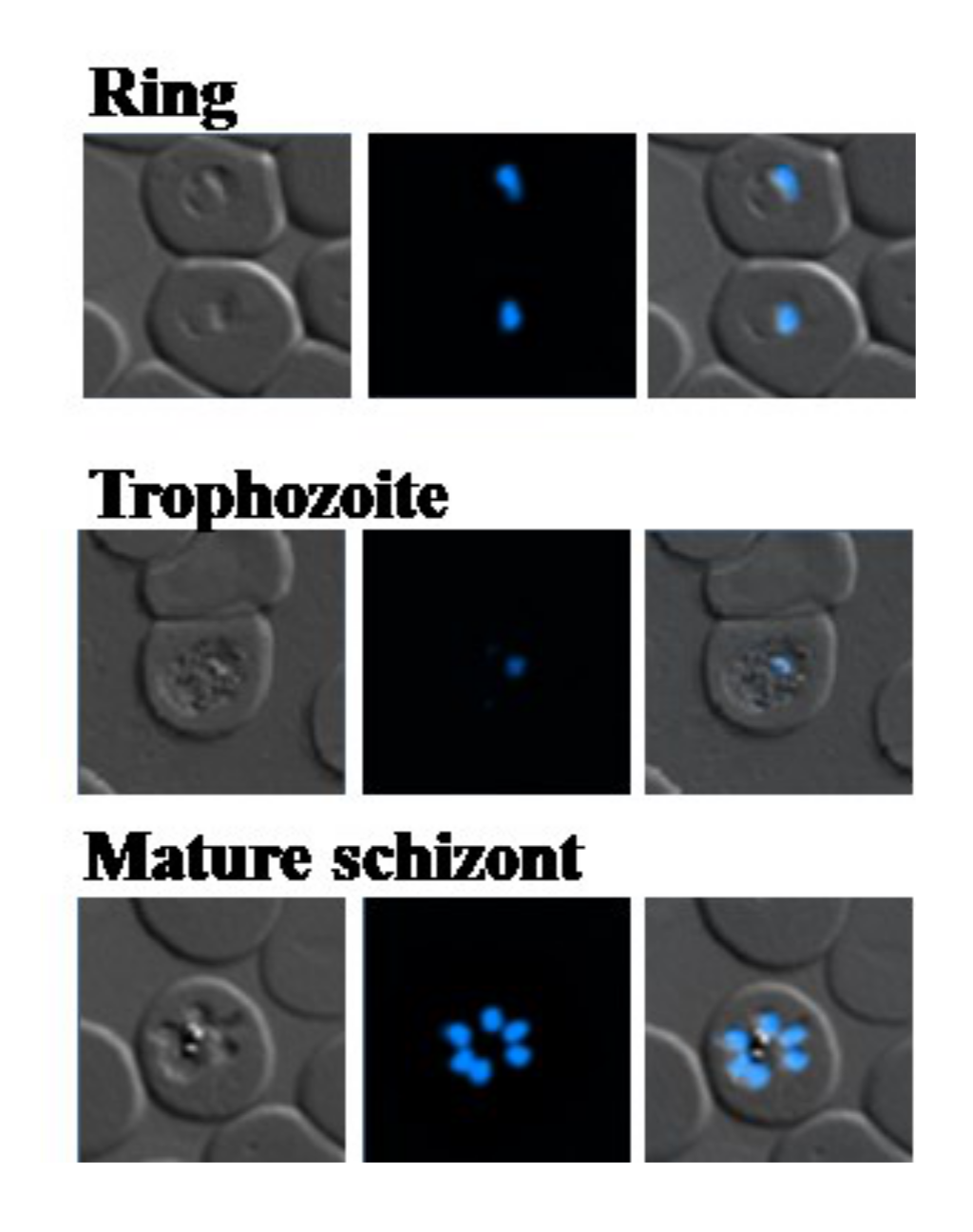

Supplement: S1 Fig — Blood smears of iRBCs were performed during merozoite development through ring, trophozoite and late schizont. Smears were fixed with 100% methanol, stained with DAPI and imaged by fluorescence microscopy and differential interference contrast with a Nikon A1 confocal microscope (objective plan Apo VC 60x, NA 1.4, λs oil immersion), and analyzed with NIS-Elements Viewer 4.20 imaging software. (TIF) [file pone.0140805.s001.tif]

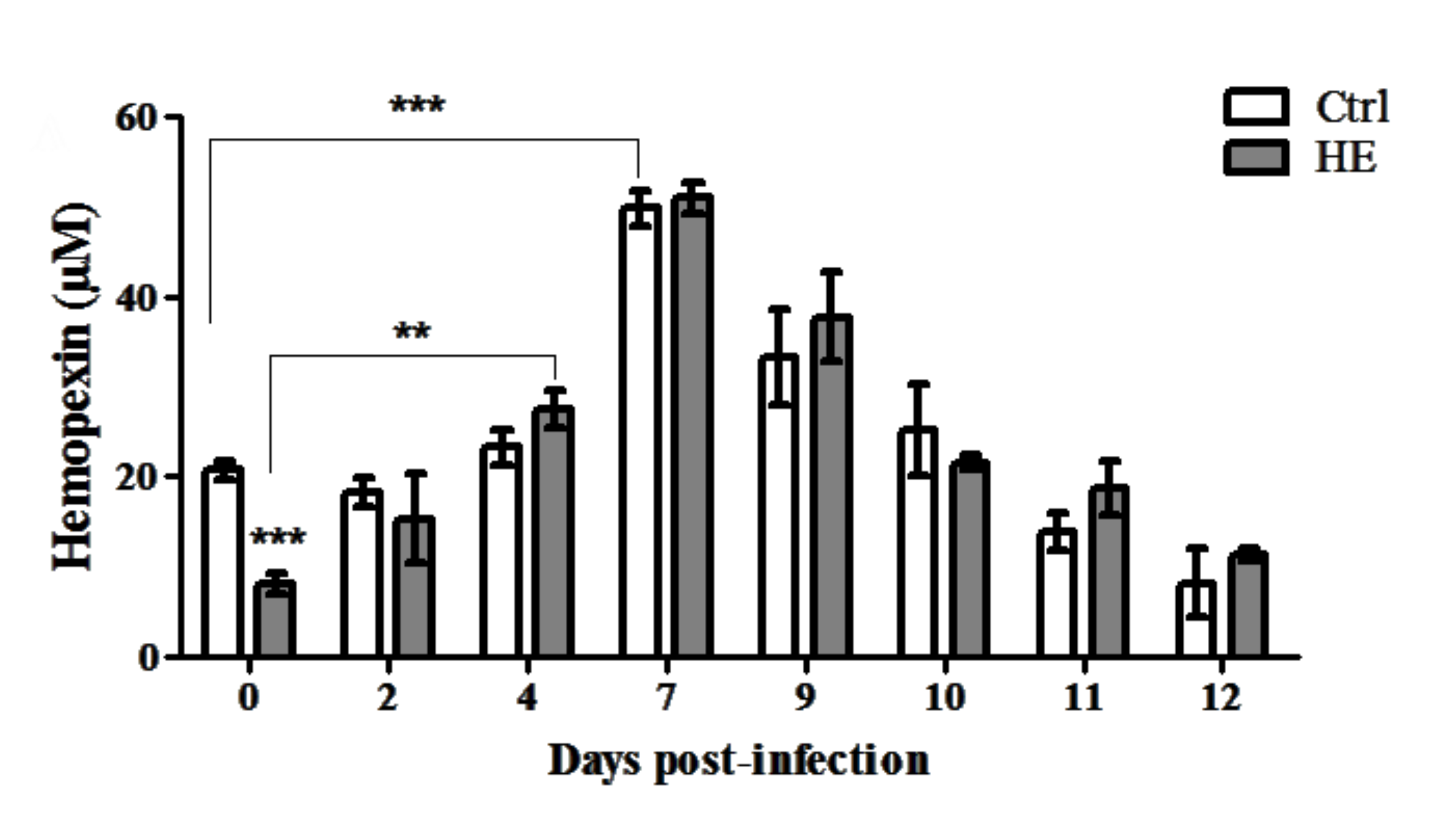

Supplement: S2 Fig — Plasmatic hemopexin levels were quantified by ELISA, 24 hours after the third saline/HE treatment, as well as throughout the infection. The results are the means ± SEM of two independent experiments (n = 3–6), and were compared with an unpaired (Ctrl/HE) and paired (Ctrl day 0/day 7; HE day 0/day4) Student t test, ** p<0.01, *** p<0.001. (TIF) [file pone.0140805.s002.tif]
